# Treatment of Acute Myeloid Leukemia with 20–30% Bone Marrow Blasts

**DOI:** 10.4084/MJHID.2013.032

**Published:** 2013-06-03

**Authors:** Luca Maurillo, Francesco Buccisano, Maria Ilaria Del Principe, Chiara Sarlo, Luigi Di Caprio, Concetta Ditto, Federica Giannotti, Daniela Nasso, Eleonora Ceresoli, Massimiliano Postorino, Marco Refrigeri, Sergio Amadori, Adriano Venditti

**Affiliations:** 1 Cattedra di Ematologia, Università “Tor Vergata”, Roma, Italia

## Abstract

The transition of patients with ≥20% <30% bone marrow (BM) blast from the FAB category of myelodysplasia to the family of acute myeloid leukemia (AML) according to the recent WHO classification has not resolved the argument as to whether the natural history and responsiveness to therapy of these diseases is comparable to that of AML with > 30% BM blast. These controversies are even more manifest when it comes to elderly patients in whom concern for intensive chemotherapy (IC) related toxicity is the critical determinant for the therapeutic choice. In fact, due to concerns of treatment-related morbidity and mortality associated with delivery of IC, approximately only 30% of all patients ≥65 years are considered eligible for this approach. Therefore, a great deal of attention has been dedicated to alternative agents such as hypomethylators (azacitidine and decitabine). Actually, these agents have shown efficacy with reduced toxicity when administered to elderly patients with 20–30% BM blasts and not eligible for IC. In the present review, we will discuss the clinical results achieved in the treatment of elderly patients with 20%–30% BM blasts AML using intensive chemotherapy (IC) or hypomethylating agents. Overall, our survey of the literature suggests that only controlled, randomized, clinical trials will answer the question as to whether hypomethylating agents has the potential to substitute for IC even in elderly patients with an optimal functional status.

## Introduction

Since 1982, “refractory anemia with excess of blasts in transformation” (RAEB-t) has been recognized as a discrete entity in the French-American-British (FAB) classification for myelodysplastic syndrome (MDS). In the FAB member’s proposal, RAEB-t should have represented an intermediate category with features transitional between acute leukemia and MDS.^1^ In fact, at the same time the FAB members also revised the definition of acute myeloid leukemia (AML) with a threshold for the diagnosis being set at 30% bone marrow (BM) blast infiltration. Based on this, a diagnosis of RAEB-t is made when blast percentage is ≥20% <30% in BM, and/or > 5% in peripheral blood (PB). Additionally, it was acknowledged that the presence of Auer rods in granulocytic precursors was consistent with the diagnosis of AREB-t irrespective of BM and PB blast count. A large number of studies have demonstrated the prognostic impact of FAB classification and the poor outlook of patients with RAEB-t whose median overall survival (OS) was about 6 months, not significantly different from that of patients with AML.^2^–^4^ Nevertheless, a number of studies have also shown that RAEB-t is a rather heterogeneous entity with the major subjects of contention being identified in the prognostic role of Auer rods and PB blast count.^5^–^8^ In 1999, the World Health Organization (WHO) published a revised classification of MDS based on which RAEB-t was abolished.^9^ Actually, because of the similarities in survival between AREB-t and AML, the panelists regarded as appropriate the reduction to 20% of the BM blast threshold for the diagnosis of AML, ratifying “de facto” AREB-t removal from MDS family. While the agreement about recognizing RAEB-1 and RAEB-2 was unanimous due to the demonstrated impact on prognosis of such a distinction,^2^–^4^ getting rid of RAEB-t was not painless. In fact, some authors argued that the inferior median OS of patients with RAEB-t was not an acceptable reason to classify and treat them as AML and suggested that clinico-biologic features other than the mere blast count might contribute to the poor outcome.^10^ The main points of controversy pertained the karyotype alterations of AML, in particular translocations, which are not so frequent in RAEB-t and the appropriateness to deliver an AML-like treatment to patients who have indolent RAEB-t and therefore might not necessitate it. Moreover, the observation that some cases of AML initially present with MDS-related features implies a previous, sub clinical MDS phase and a pathogenesis different from other cases of AML lacking dysplasia. Therefore, the 3rd edition of WHO classification included a new AML subtype called “AML with multilineage dysplasia” (AML-MLD).^11^–^12^ In this category, patients were included with 20% or more blasts, and with: 1) morphologic dysplasia in 50% or more of at least two cell lineages, or 2) evolution from a previous MDS or myelodysplastic/myeloproliferative (MDS/MPN) neoplasm. The creation of this new category mitigated only in part the disappointment resulting from the elimination of RAEB-t. Indeed, some authors assume that the prognostic power of such a category is questionable if based only on features of multilineage dysplasia; therefore they suggest that disease outcome is better established when multilineage dysplasia (MLD) is associated with genetic abnormalities.^13^–^14^ Conversely some other authors emphasize the clinical utility of this category even when defined by the sole morphology.^15^ As a matter of fact, WHO now includes in this category also patients with specific myelodysplasia-related cytogenetic abnormalities regardless of whether or not multilineage dysplasia is morphologically documented (“AML with myelodysplasia-related changes”, AML-MRC).^16^

Finally, argument still remains as to whether the natural history and responsiveness to therapy of patients with 20%–30% BM blasts is comparable to that of patients with >30% BM blast AML.

Based on this background, we will discuss in the present review the clinical results achieved in the treatment of patients with 20%–30% BM blasts AML using intensive chemotherapy (IC) or hypomethylating agents.

## Cytogenetics and Genetic Abnormalities

In 2008, the WHO classification created a new category called AML-MRC with or without morphological MLD associated. By designing this category which encompasses cases of AML characterized by MDS-like features, the experts tried to work-out the issue of RAEB-T elimination since the vast majority of AML-MRC present with a 20% to 30% BM blasts infiltration. A further element of interest relates to the observation that MLD features are frequently associated with an adverse karyotype.^13^–^14^,^17^ A recent German study, compared 408 adult patients diagnosed as AML-MRC or AML not otherwise specified (NOS). MLD pattern significantly correlated with pre-existing MDS and MDS related cytogenetic changes. AML NOS and AML-MRC associated with MLD features had a superior EFS and OS compared with AML-MRC diagnosed for history of MDS or for MDS related cytogenetics.^17^ Table 1 report the cytogenetic abnormalities needed for a diagnosis of AML-MRC when 20% or more PB or BM blasts are present: although balanced chromosomal aberrations can be detected, these abnormalities include mainly unbalanced karyotype lesions. Patients who are categorized in this subgroup and have a normal karyotype should be analyzed for FLT3, NPM1, and CEBPA mutations, frequency of which is approximately 20%, 30% and 9%, respectively, in de novo AML-MRC with MLD features and approximately 7%, 12% and 7%, respectively, in AML-MRC supervening after a previous history of MDS or with MDS related cytogenetics.^14^,^17^ As a consequence of such a complex scenario some authors proposed to omit the group of patients classified as AML-MRC only on the basis of MLD features.

The close biological and clinical relationship between AML with 20–30% of BM blasts and high risk MDS, including the efficacy of hypomethylating agents and the poor response to conventional chemotherapy, leads to the hypothesis that epigenetic deregulation might be implicated in their pathogenesis. A series of single locus studies have demonstrated that several genes are silenced in association with the methylation of their promoter. These include genes participating into cell-cycle regulation, apoptosis, adhesion and motility, and other pathways. Among these, CDKN2B (p15) hypermethylation is frequently found in RAEB-t or AML supervening after MDS^18^–^19^ and is associated with old age, deletions of 5q and 7q, and a poor prognosis.^20^ Such a hypermethylation was calculated in rates from 0 % in low-risk MDS, to 30 % in high-risk MDS, up to 75 % in AML transformed from MDS.^19^ Recent advances in technologies such as high-resolution single nucleotide polymorphism (SNP) array and next-generation sequencing have led to the identification of somatic mutations in epigenetic as well as post-translational histone modifications regulators.^21^–^22^ Somatic mutations affect genes that encode proteins regulating DNA cytosine methylation, hydroxymethylation and demethylation. DNA methyltransferase 3A (DNMT3A) mutation is thought to promote gene hypomethylation but it is not yet known which genes are altered. Its frequency in AML is approximately 12–22% and is associated with poor prognosis. Mutations in Tet methilcytosine deoxygenate 2 (TET2) alter conversion of 5 methylcytosine (5-mC) to 5-OH-methylcytosine (5-hmC), an intermediate event producing gene demethylation. In AML, it occurs at an estimated frequency of 7–23% and it is associated with poorer prognosis in patients with favorable cytogenetics or cytogenetically normal AML (CN-AML). Mutations in isocitrate dehydrogenase 1/2 (IDH1/2), result in a neomorphic, 2-hydroxyglutarate-driven enzymatic activity which serves as a competitive inhibitor of TET family of enzymes. Studies of a large cohort of AML patients demonstrated that IDH mutations frequency is 15–33% and that IDH and TET2 mutations are mutually exclusive. Yet, results on prognostic effects in AML are divergent.

Post-translational histone modifications cause mutations in histone-modifying enzymes. Enhancer of Zeste Homolog-2 (EZH2) is the main member of Polycomb group (PcG) proteins which are transcriptional repressors regulating cell differentiation. Mutations of EZH2 are rare in AML and are associated with a worse OS in MDS. It is unclear whether mutations of Addition of Sex Combs-like 1 (ASXL1) confer a loss or a gain of function. It has recently been suggested that ASXL1 loss of function results in disarrangement of the transcriptionally repressive H3K27 thrymethylation with consequent increase of HOXA gene expression. ASXL1 mutations frequency is approximately 5% in AML and it is associated with poor prognosis. Finally, Mixed-Lineage Leukemia (MLL) is a member of a multiprotein complex that mediates the methylation of H3K4 within the promoter region of genes. MLL can be affected by mutations that result in partial tandem replication (PTD) which in turn can boost the levels of H3K4me3 at the level of target genes. MLL-PTD frequency is 4–7% and is associated with poor prognosis in AML and CN-AML. MLL is also involved in translocations at the locus (11q23); such alterations are frequent in infant leukemia and therapy-related AML (frequency 10–15%). This translocation is frequently linked with acquisition of H3K79 methyltransferase activity.

## Comorbidites and Treatment Choice in Elderly Patients with AML

Although patients aged ≥60 years are prone to experience greater treatment related toxicity than younger subjects and, therefore, to have a shorter overall survival as a consequence of such toxicity, there is evidence that selected older adults can benefit from intensive chemotherapy delivery. Based on this, it became clear that age alone may be an inadequate measure of patients’ eligibility to intensive treatments, whereas Karnofsky and ECOG PS scales are subjective and may lack sensitivity in capturing meaningful impairments in physical functions.^23^ Based on this, multidimensional geriatric assessments have been developed by interrogating host-specific clinical characteristics such as cognitive, physical and psychological function with the aim to predict vulnerability to chemotherapy. Such an approach, more reliably than PS scales, allows for those who should be addressed to alternative strategies (e.g. hypomethylating agents) to be identified. An exploratory experience demonstrated a clear-cut correlation between geriatric assessment and tumour aggressiveness and those cognitive, psychological and physical impairments were observed across all cytogenetic subsets.^24^ A further step in this line of research is to evaluate, prospectively, the correlation between such a risk assessment, survival parameters, treatment toxicity and early mortality in order to implement approaches of individualized treatments.

## Intensive Chemotherapy

AML is a common disease of the adult age with a peak incidence between 65 and 70 years. According to recent epidemiological data, ≥75% of patients with AML are ≥60 years old.^25^ In this age-range, AML is an incurable disease with less than 10% of patients being alive at 2 years.^26^ Such a dismal outcome has been traditionally explained by the concurrence of co-morbidities and biologically poor-risk AML features. Although co-morbidities can hamper delivery of IC, and in spite of reluctance of physicians to expose older patients to the toxic effects of the anti-leukemic therapy, population-based studies have demonstrated that IC prolongs survival and ameliorates quality of life in all age groups, as compared to palliative therapy.^27^ Nevertheless, only about one-third of elderly patients receive IC^28^ with low-dose cytosine arabinoside (ARAc), farnesyl inhibitor tipifarnib and gentuzumab ozogamicin (GO) being identified as potential alternatives.^27^ As a consequence of this situation, the optimal remission induction and post-consolidation therapy in elderly patients has not yet been determined. In this section we will review the most recent advances in standard treatment of elderly AML with a special focus on patients with 20%–30% BM blast count. In all of the considered studies, diagnosis of AML grounded on WHO criteria. However, since the amount of BM blast infiltration did not emerge as a prognosticator either in univariate or multivariate analysis, we assume that most of the clinical results reported below may be extended to the specific subset of elderly patients with 20%–30% BM blast. This is in line with the results of two seminal studies by Estey^29^ and Bernstein,^30^ either agreeing that a diagnosis of RAEB-t or AML did not have any impact on the effect of IC. Estey and coworkers treated 106 patients affected with RAEB-t and 372 with AML by delivering a combination of high dose ARAc, idarubicine (IDA) and fludarabine or topotecan. Complete remission (CR) rate and event free survival (EFS) were identical (66% and 7 months, respectively; P=NS for both). Bernstein and coworkers, treated 33 patients with RAEB-RAEB-t according to 1984–1992 CALGB AML protocols achieving similar outcomes in terms of CR rate (79% and 68%, P=NS) and EFS (11 and 15 months, P=NS).

### Induction intensification

Table 2 summarizes the most recent studies exploring the effect of IC in elderly AML. In all of the studies, eligibility criteria required at least 20% BM blast infiltration with the exception of National Cancer Research Institute (NCRI) and HOVON-SAKK studies in which also patients with >10% BM blasts were included. Lowenberg has recently reported on a randomized trial comparing high-dose (90 mg/m^2^ for three days) versus conventional dose (45 mg/m^2^ for three days) daunorubicin (DNR), given as an induction regimen in association with standard-dose ARAc. The study recruited 813 elderly patients with previously untreated AML.^31^ Despite a significantly superior CR rate in the high-dose DNR arm (64% vs 54%, P=.002), no difference was observed in terms of OS and EFS. Induction death rate was similar (11% vs. 12%, P=NS). A post-hoc analysis demonstrated that the patients who benefited the most by escalated DNR were those aged 60 to 65, and that the beneficial effect took place in terms of CR frequency (73% vs 51%) and 2-year EFS and OS duration (29% vs 14%, P=.002 and 38% vs 23%, P<.001, respectively). Increasing age, poor PS, presence of splenomegaly or extramedullary disease, high white blood cell count (WBCc) and unfavorable cytogenetic were associated with a short duration of OS and EFS. However, no impact of BM blast infiltration on overall outcome was demonstrated. The results of the HOVON-SAKK study were not confirmed in the ALFA-9801 study.^32^ The backbone of this protocol was the comparison between high-dose DNR (80 mg/m^2^ for 3 days) versus standard-dose (12 mg/m^2^ for 3 days) or escalated dose (12 mg/m^2^ for 4 days) of IDA in 468 AML patients aged 50–70 years. Neither high-dose DNR nor high-dose IDA gained any significant superiority over IDA given at a standard dose. In multivariate analysis, the age of 60 years came out as an independent prognosticator for OS, WBCc and cytogenetics for OS and EFS; induction death rate was similar across the two treatment arms (6% vs 8% vs 6%, P = NS, respectively). The NCRI and HOVON-SAKK experience indicates that anthracycline dose escalation is feasible and does not endanger elderly patients more than conventional approaches; however definitive advantage in terms of OS and EFS is not yet demonstrated.

### New agents as monotherapy or combination therapy

The combination of standard chemotherapy and multi-drug resistance (MDR) modulators has been explored in large international trials. Nevertheless, both the NCRI AML14 trial and the ECOG 3999 trial failed to demonstrate any advantage from the administration of the MDR modulators PSC833 or Zosuquidarin combination with chemotherapy.^33^–^34^ The lack of benefit of these agents suggested that they were either not effective inhibitors of MDR or that MDR inhibition, even if occurring, was not enough profound to overcome drug resistance. Clofarabine, administration of which is approved for patients with acute lymphoblastic leukemia, has recently been tested in patients with relapsed or refractory AML, as well as in elderly patients with previously untreated AML.^35^–^36^ As a single agent, Clofarabine 30 mg/m^2^ for 5 days, was challenged in 2 clinical trials (UWCM001 and BIOV-121) which enrolled an overall number of 106 elderly patients. Overall response rate (ORR) was 48% with an early death rate of 18%. In either the studies, the authors observed that in patients with adverse risk cytogenetic, who accounted for approximately one third of the whole population, ORR was similar to the other cytogenetic risk group. Clofarabine was also tested in combination with other drugs, especially ARAc and hypomethylating agents.^37^ Clofarabine 20 mg/m^2^ for 5 days and subcutaneous ARAc 20 mg twice daily for 10 days achieved an encouraging response rate of 66%. Median OS and relapse free survival (RFS) was 12.7 and 14.1 months, respectively, median OS of responding patients was 24.2 months and induction mortality low (7% at 8 weeks) with manageable toxicities. Upcoming randomized phase 3 trials will compare clofarabine and standard induction therapy in elderly patients with AML. The postulated activity in poor prognostic groups such as those of patients with adverse cytogenetic makes this drug particularly attractive for the management of patients with low BM blast count, therefore post-hoc analysis of this category will be very welcome once the above mentioned protocols are closed.

### Post-remission therapy

Once CR is achieved, there is no consensus about intensity and duration of post-consolidation treatment. This topic has never been addressed in prospective randomized trials; however some conclusion may be deduced by the analysis of the largest published studies. Neither high-dose ARAc^38^ nor post-remission administration of GO^39^ or interleukin-2^40^ have been demonstrated to be of benefit in this population. Three major studies have tried to evaluate duration and types of post-remission therapy (Table 3). NCRI-AML11 trial demonstrated that 4 intensive consolidation cycles added no survival advantage when compared to 1 cycle of moderate intensity chemotherapy.^41^ ALFA9803 and AML-HD98 reached divergent conclusions. The former^42^ demonstrated a benefit in terms of OS (56% vs 37%, P=0.030) and disease free survival (DFS) (28% vs 17%, P=0.040) for elderly patients receiving 6 months of low-dose therapy as compared with those undergoing a single intensive consolidation cycle. At the opposite, HD98 study^43^ showed a survival benefit for those patients receiving 2 intensive multi-drug consolidation therapies as compared with those treated by mild oral maintenance (median OS 22.3 vs 14.3 months, P=0.04). These discrepant results may be explained by the different composition of the patients’ population in the AML-HD98 study such as the over-representation of intermediate risk karyotypes and the few patients randomized in the consolidation arm. Actually, a major drawback of the clinical trials involving elderly patients consists in the high withdrawal rate that results in a less than expected number of patients receiving the assigned therapy. This may be troublesome when it comes to data analysis and interpretation even if intent to treat approach is used. It is our opinion that consolidation cycles should be administered to all eligible patients even if the randomized trials have not provided the formal and definitive demonstration of the superiority of such an option. In line with this, recent reports suggest that, for patients aged 60–65 years, reduced intensity stem cell transplantation (RIC) represents the logical and natural extension of the eligibility to intensive chemotherapy. In these reports, it was proven that age up to 70 years doesn’t seem to affect the outcome of transplant so that RIC can be regarded as a valid option in this category of patients.^44^–^46^ The largest series published to date entail data from 1036 AML patients aged 50 to 70 years who were in first CR. Of these 1036 patients, 152 underwent allogeneic SCT and 884 were given consolidation chemotherapy. In the group treated by transplant, the cumulative incidence of relapse was significantly lower (22% vs 62%) and OS significantly longer (62% vs 51%, P=.012) than in that of patients receiving consolidation courses.^47^

## Hypomethylating Agents

IC may not be the most appropriate option for elderly patients who have a poor performance status, co-morbid conditions, deficient BM reserve and for whom a significant treatment-related morbidity and mortality can be anticipated.^41^,^48^–^49^ These observations boosted search for alternative therapies that, although less intensive, could retain the potential of inducing CR, prolonging survival, and preserving quality of life. Emerging research has showed that silencing of key genes critical to growth, differentiation, angiogenesis, signalling, and DNA repair is mediated by aberrant DNA hypermethylation and/or histone deacetylation.^50^–^52^ Aberrant DNA methylation has been demonstrated to occur in stem cell compartment of MDS and AML,^53^ and, since it is believed to be reversible, it represents an attractive therapeutic target for DNA methyltransferase (DNMT) inhibitors such as azacitidine and decitabine.^54^

### Azacitidine

Azacitidine is a pyrimidine nucleoside analogue of cytidine which is phosphorilated intracellularly to generate its active form azacitidine triphosphate; this phosphorylation is mediated by uridine-cytidine kinase. In turn, azacitidine is incorporated either into RNA, interfering with the synthesis of nucleic acids and proteins and, to a less extent, into DNA.^55^–^56^ The drug inhibits DNMT, therefore inducing hypomethylation and transcription of quiescent genes, restoring cancer-suppression functions and induction of cellular differentiation. Finally, there is evidence of a direct cytotoxic effect on BM abnormal hematopoietic cells.^57^

This drug has been approved in United States (USA) for treatment of MDS of all FAB-defined subtypes and in the European Union (EU) for the treatment of patients not eligible for hematopoietic stem cell transplantation with: int-2- or high-risk MDS; chronic myelomonocytic leukemia with 10–29% bone marrow blasts without myeloproliferative disease; and WHO-classified AML with 20–30% blasts and multilineage dysplasia.

The evidence of the efficacy of azacitidine in patients with 20–30% BM blast AML and multilineage dysplasia comes from two large phase III studies and from registry programmes for compassionate-use surveys.

#### Phase III clinical trials

The phase II Cancer and Leukemia Group B (CALGB) trials (CALGB 8421 and 8921) demonstrated the efficacy of intravenous (iv) and subcutaneous (sc) azacitidine in patients with FAB-defined MDS.^58^ The following phase III trial (CALGB 9221) compared sc azacitidine with supportive care in patients with MDS diagnosed according to the FAB classification criteria.^59^ Overall, 309 patients were included in the 3 studies; 268 were treated with azacitidine (220 received sc azacitidine at the dose of 75 mg/m^2^ daily for 7 consecutive days) and approximately 60% of them were ≥65 years old. When WHO criteria were applied, 103 patients were reclassified as AML: 25 in CALGB 8421, 26 in CALGB 8921, 52 in CALGB 9221 (27 in the azacitidine arm, 25 in the supportive care arm, 13 of whom were treated with azacitidine after the observation phase), for a total of 91 patients treated with azacitidine. By using the “International Working Group” (IWG) response criteria for MDS,^60^ among the 91 patients treated with azacitidine the ORR (CR + partial remission + haematological improvement) was 36% and the median duration of response 7.3 months (range, 2.2 to 25.9 months). When the analysis was focused on the phase III trial CALGB 9221, the median OS of patients in the azacitidine arm was 19.3 months vs 12.9 months of those in the supportive care arm (Table 4). Although CR rate was lower (9%) than what expected with IC, the median OS of 19.3 months was comparable to that obtainable with chemotherapy, suggesting that azacitidine may alter the natural history of the disease regardless of the achievement of CR. Additionally, there was no increase of infections or haemorrhage in the azacitidine group beyond what expected for AML patients.^61^

Because the survival advantage with azacitidine was established in patient subgroups (higher-risk patients) or after a landmark analysis to eliminate the confounding effect of the crossover design of the CALGB-9221 study, an additional trial was designed to confirm the OS benefit. The AZA-001 trial was an international, randomized, phase III study of azacitidine 75mg/m^2^/day for 7 days of each 28-day cycle, for at least 6 cycles, compared with the three most common conventional regimens (CCR) in Int-2 and high-risk MDS; prior to randomization, investigators were required to preselect patients to the CCR considered most appropriate: the choice was between supportive care (BSC), low-dose ARAc (LDAC) 20 mg/m^2^ for 14 days every 28 days for at least 4 cycles, or IC consisting of an induction with ARAc 100–200 mg/m^2^/day for 7 days plus 3 days of DNR 45–60 mg/m^2^/day or IDA 9–12 mg/m^2^/day or mitoxantrone 8–12 mg/m^2^/day. 62 A total of 358 patients were included in the study and 113 (30%) had a diagnosis of AML with trilineage dysplasia (Table 4). Of these 113, 55 were assigned to the azacitidine arm and 58 to the CCR (27 BSC, 20 LDAC and 11 IC). Median age of those treated on azacitidine was 70 years (range 52–80), median BM blast percentage was 23% (range 20–34%), 69% and 25% of the patients had intermediate and adverse cytogenetic risk group, respectively. The CR rate in azacitidine, LDAC and IC arm was 18%, 15% and 55%, respectively. Median OS in the azacitidine and CCR groups was 24.5 and 16.0 months, respectively (P=.004) and the 2-year OS rates were 50% and 16%, respectively (P=.001). When the analysis was broken down according to each CCR group, median OS was significantly superior in the azacitidine group versus BSC (19 vs 13 months, respectively, P=.03) but no survival advantage was found versus LDAC group (median overall survival 24.5 vs 17.0 months, respectively) or IC group. Results from comparison of azacitidine versus IC should be considered with extreme caution because the IC group accounted only for 11 patients. The AZA-001 trial provided some evidence of azacitidine-associated benefits. In fact, azacitidine prolongs survival despite a lack of response; it is very well tolerated with nearly all adverse events occurring during the first two cycles of treatment and the rate of infections and hospitalization are significantly lower than in patients receiving CCR.^62^–^63^ However, it should also be considered that the median OS in the CCR arm was atypically long therefore suggesting bias in patient selection. In particular, the exclusion of patients with high WBCc-AML, who are known to have a worse prognosis, sounds critical. In order to answer this issue, a phase III trial is currently being conducted in patients aged ≥65 years, with untreated de novo or secondary AML.^64^ A subsequent AZA-001 post-hoc analysis focusing on patients aged ≥75 years confirmed the previous finding of an OS advantage for the azacitidine arm (table 4).^65^

#### Compassionate use of azacitidine

In addition to data from phase III studies, early trials and registry programs can help extrapolate the efficacy of azacitidine in patients with AML. In the French azacitidine compassionate use program (ATU), azacitidine was given to previously untreated or relapsed/refractory AML patients in the same dose and schedule as in the AZA-001 trial. In a first retrospective analysis (Table 4), 138 elderly untreated patients with a median age of 73 years were evaluated. Of these 138, 44 (32%) had 20–29% BM blasts infiltration, 61 (44%) had an adverse karyotype; 65 (47%) and 30 (22%) had secondary and therapy related AML, respectively. ORR and CR were 21% and 14%, respectively; 2-year survival was 18%, median survival 10.2 months. Unfavourable karyotype and a WBCc > 10×10^9^ leukocytes/L but not the percentage of BM blasts, were associated with a poor prognosis.^66^ A second retrospective analysis included 184 patients with relapsed/refractory AML: median age was 64 years, 15 (8%) patients had 20–29% BM blasts infiltration, 55 (30%) an adverse karyotype, 57 (31%) and 15 (8%) secondary and therapy related AML, respectively. ORR and CR were 11% and 7%, respectively; 1-year survival was 29.1%, median OS 7.8 months.^67^ In a recent analysis carried out in 282 patients treated with azacitidine, with high or intermediate-2 risk MDS and AML <30% blasts (22% of the series = 62), factors independently associated with lower response rates were previous LDAC treatment, BM blasts >15%, and cytogenetic abnormalities. Complex karyotype predicted a significantly shorter duration of response whereas performance status >2, intermediate- and poor risk cytogenetics, presence of circulating blasts and red blood cell transfusion-dependency >4 units/8 weeks were independent factors associated with a shorter duration of OS.^68^ An Italian retrospective study on 82 patients with AML treated with azacitidine in a compassionate program setting, showed similar results (table 4); in fact, in 35 untreated AML patients (46% of them with 20–29% of BM blasts), ORR and CR were 48% and 19%, respectively, 2-year survival 13% and median OS 9 months; in multivariate analysis, the chance of obtaining a response was significantly associated with a WBCc < 10×10^9^/L. In 47 patients with pre-treated AML (36% of them with 20–29% of BM blasts), ORR and CR were 19% and 11%, respectively, 2-year survival 7%, median survival 7 months. 69 Other studies suggested that a diagnosis of de novo AML and BM blast count ≤45% on day 15 of the first treatment cycle^70^ or platelet doubling after the first azacitidine cycle^71^ are useful predictors of response to azacitidine.

#### Combination therapy with azacitidine

Several combinations of azacitidine with other agents have been explored in order to improve results obtained with azacitidine monotherapy. To date, no randomized trial has compared the efficacy of azacitidine in combination versus azacitidine alone. Azacitidine has been challenged in combination with valproic acid, ATRA,^72^–^73^ or thalidomide^74^ but ORR and CR (50% and 20%, respectively) were not significantly different from those achieved using azacitidine monotherapy. Association therapy of azacitidine and the histone deacetylase inhibitor vorinostat also exhibited promising activity in patients with AML. In a phase I trial conducted in 28 patients with AML and MDS, 10 (83%) of the 12 with AML/high-risk MDS reached a CR.^75^ Combination of azacitidine and lenalidomide is another approach eagerly investigated. Forty-two untreated patients with a median age of 74 years (range 62–86) received azacitidine 75mg/m^2^ for 7 days followed by escalating doses (5 mg, 10 mg, 25 mg and 50 mg) of lenalidomide daily for 21 days starting on day 8 of a 28-day cycle. Patients remained on therapy until disease progression, unacceptable toxicity or completion of 12 cycles. ORR was 40% (CR + CRi rate 28%). Duration of CR/CRi was 28 weeks (range, 4- >104 weeks), therapy-related acute myeloid leukemia and high comorbidity index anticipated a short duration of response.^76^ More recently, phase II studies were carried out to explore the role of azacitidine as a maintenance therapy after conventional IC. In a first trial, 23 out of 60 AML/high risk MDS patients who achieved CR after a cycle of DNR plus ARAc, were given azacitidine at 60 mg/m^2^ for 5 days every 28 days until relapse or unacceptable toxicity. The median age was 68 years, the median duration of response was 13.5 months and in 30% of the patients a sustained CR, lasting longer than 20 months, was observed.^77^ In a second trial, 46 patients (33 AML and 13 high-risk MDS) were included. The median age was 66 years, azacitidine was delivery at a dose of 60–75 mg/m^2^ for 5 days every 28 days and the median number of cycles administered was 5.5. Thirty-three patients achieved CR/CRi and two partial remission, DFS and OS rate at 18 months were 30% and 56%, respectively and median OS was 24 months. In the authors’ experience, OS and DFS of these subsets of patients were comparable to a similar cohort of AML patients treated with a second consolidation course of anthracyclines and Ara-C.^78^

### Decitabine

Although even decitabine (5-aza-2′-deoxycytidine) is a pyrimidine analogue of cytidine with inhibitory effects towards DNMT, it characterizes for some pharmacokinetic differences compared to azacitidine. Once given iv, it undergoes a rapid deamination so that its half-life is relatively short. Cellular uptake of decitabine takes place through a nucleoside transport mechanism and, once into the cellular environment, it is phosphorylated by deoxycytidine kinase into 5-aza-2′-deoxycytidine-5′-triphosphate. Decitabine is incorporated exclusively into DNA where it binds covalently DNMT1 therefore depleting the cells of this enzyme. At low doses, decitabine can cause AML cell lines to differentiate and enter apoptosis^79^ whereas at high concentrations it has a cytotoxic effect, possibly leading to generation of alkali-labile DNA strands. In USA and EU, decitabine is approved for the treatment of previously treated and untreated, de novo and secondary MDS of all FAB subtypes and intermediate 1/2 and high-risk IPSS groups.

#### Phase II- III clinical trials

Clinical trials investigating the role of decitabine as a first-line therapy in newly diagnosed AML of elderly patients are underway; available data from concluded clinical trials are summarized in table 5.

An initial multicenter phase II study of low dose of decitabine was conducted in elderly patients with newly diagnosed AML.^80^ The drug was administered intravenously at the dose of 20 mg/m^2^/day for 5 days, every 4 weeks. Median age was 74 years and 18 (33%) patients had BM blasts <30%. As a whole, ORR and CR rate were 25% and 24%, respectively. The authors observed a slight increase of CR rate to 28% when patients with BM blasts <30% were extrapolated. Similarly to azacitidine studies, even in this decitabine trial no responses were observed in patients with a peripheral absolute blast count greater than 10×10^9^/L. Median OS was 7.7 months and subgroup analysis demonstrated that patients with stable disease had an OS longer than the one of no responders. The most common toxicities, myelosuppression, febrile neutropenia and fatigue, were all manageable. Of interest, the efficacy of decitabine was not compromised in patients with poor-risk cytogenetics, a subgroup often associated with inferior outcomes.

In another study, 53 elderly patients, not candidates for or refusing IC, received an induction 10-day regimen of decitabine at 20mg/m^2^ followed by 4–5 day cycles.^81^ Median age was 74 years (range 60–85 years), although the median BM blast infiltration was 52% (range 20–92%) an exact estimate of patients with BM blasts <30% was not retrievable from the study. With a median of 3 delivered cycles of therapy, the CR rate was 47% and subgroup analyses demonstrated comparable CR rates regardless of age, cytogenetics, presenting WBCc and whether the AML was de novo or evolved from an antecedent hematologic disorder. Median overall survival was 12.7 months. The clinical study was also paralleled by a translational research which documented that the higher the levels of miR-29b the more frequent the responses to decitabine. A multicenter, randomized phase III trial comparing decitabine versus investigator’s treatment choice (TC = supportive care or LDAC) enrolled 485 elderly patients with AML.^82^ Approximately one fourth of the patients had BM blasts <30%, 71% were ≥70 years of age, 35% had secondary AML and 36% had poor-risk AML. In those randomized to the experimental arm, decitabine was given at the dose of 20 mg/m^2^ per day as a 1-hour iv infusion for five consecutive days, every 4 weeks. Although decitabine arm achieved a significantly higher CR rate compared to TC arm (17.8% vs 7.8%, respectively) at the planned clinical cutoff date no differences in terms of OS were seen (7.7 vs 5 months, respectively). Only a subsequent post hoc analysis came out with a significant better OS in favor of patients treated in decitabine arm (P =.037).

#### Combination therapy with decitabine

Studies using decitabine in association with other agents in patients with AML are few and very preliminary; therefore, firm conclusions cannot be drawn. In the attempt to silence multiple pathways involved in epigenetic changes, decitabine at 20 mg/m^2^ daily for 10 days or at 20 mg/m^2^ daily for 5 days has been given together with escalating doses of valproic acid or vorinostat. This strategy gained ORR of 44% and 36%, respectively.^83^–^84^ Still ongoing clinical trials include decitabine in combination with bexarotene (NCT01001143), midostaurin (NCT01130662), bortezomib (NCT01420926) and sapacitabine (NCT01303796). A Phase 3, open-label, randomized clinical trial is recruiting elderly patients with newly diagnosed AML to receive either ARAc/anthracycline-based induction regimen followed by ARAc consolidation therapy or clofarabine single agent as induction and consolidation therapy. Patients are then randomized to decitabine maintenance therapy for 12 months or observation (NCT01041703).

## Conclusions

Overall, the current literature indicates that AML outcome in elderly patients remains disappointing. Physicians’ anxiety about treatment-related morbidity and mortality potentially associated with delivery of IC causes that approximately only one third of all patients aged ≥65 years are considered eligible for this approach. Moreover, the percentage of treated patients decreases progressively with each advancing decade of life, with only 6% of patients over the age of 85 receiving IC. Therefore, in recent years an ever more growing attention has been paid to development of effective and tolerable therapies for elderly patients. In this view, the discovery of the hypomethylating power of old drugs has led to the hope of cure with minimal toxicity for specific categories of elderly patients such as those not eligible for IC and with 20–30% BM blasts plus multilineage dysplasia. The clinical trials of azacitidine have demonstrated the feasibility of an outpatient administration and the favourable toxicity/efficacy ratio with lower incidence of complications and hospital admissions compared to IC. At the same time, we are not yet in the position to recommend that hypomethylating agents substitute for IC in elderly patients with an optimal functional status. This issue will hopefully be addressed in future controlled, randomized clinical trials.

## Figures and Tables

**Table 1:** Cytogenetic abnormalities sufficient for diagnosis of AML-MRC.

Complex karyotype*	Unbalanced abnormalities	Balanced abnormalities

	−7 or del(7q)	t(11;16)(q23;p13.3) **
	−5 or del (5q)	t(3;21)(q26.2;q22.1) **
	i(17q) or t(17p)	t(1;3)(p36.3;q21.1)
	−13 or del(13q)	t(2;11)(p21;q23) **
	del(11q)	t(5;12)(q33;p12)
	del(12p) or t(12p)	t(5;7)(q33;q11.2)
	del(9q)	t(5;17)(q33;p13)
	idic(X)(q13)	t(5;10)(q33;q21)
		t(3;5)(q25;q34)

* Three or more unrelated abnormalities, none of which are included in the “AML with recurrent genetic abnormalities” subgroup; such cases should be categorized in the appropriate cytogenetic group.

** Abnormalities that most commonly occur in “therapy-related AML”: the latter should be excluded before using such abnormalities as evidence for diagnosis of AML with myelodysplasia-related changes.

**Table 2:** Intensive regimens for elderly AML

Study	Age (range)	N∘	Induction and post-remission	CR	ED	OS
HOVON- SAKK^31^	67 (60–83)	813	*INDUCTION* ARAc 200 mg/m^2^ + DNR (45 mg/m^2^ vs. 90 mg/m^2^) × 3 days ARAc 1g/m^2^ q 12 h × 6 days *POSTREMISSION* Allogeneic SCT or GO or no treatment	P=0.002 54% vs 64%	P = NS 12% vs 11%	30% (2-yrs)
ALFA-9801^32^	60 (50–70)	478	*INDUCTION* DNR 80 mg/m^2^× 3d vs IDA 12 mg/m^2^ ×3d vs IDA 12 mg/m^2^ × 4d +ARAc 200 mg/m^2^× 7 days *POSTREMISSION* ARAc 1 g/m^2^ q 12 h × 4d + induction anthracycline × 2d	P = NS 70% vs 83% vs 78%	P = NS 8% vs 3% vs 6%	38% (2-yrs)
ALFA-9803^42^	72 (65–85)	429	*INDUCTION* DNR 45 mg/m^2^ × 4d or IDA 9 mg/m^2^ × 4d + ARAc 200 mg/m^2^× 7 days *POSTREMISSION* ARAc 1 g/m2 q 12 h × 5d + induction anthracycline ×1d for 6 months vs ARAc 200 mg/m^2^ × 7d + anthracycline × 4d	57%	10%	27%
AML HD98^43^	65 (61–78)	329	*INDUCTION* ICE 2 cycles +-ATRA *POSTREMISSION* HAM 1 cycle Random IEiv 1 cycle or 1-year oral maintenance therapy IEpo	46%	-	24% (4-yrs)
AML14^33^	67 (44–88)	1273	*INDUCTION* DAT × 2 courses +-PSC833 *POSTREMISSION* Random: MIDAC ± ICE	54%	18%	13% (5-yrs)

Abbreviations: CR = complete remission; ED = early death; OS = overall survival; ARAc = cytosine arabinoside; DNR = daunorubicine; SCT = stem cell transplantation; GO = gemtuzumab ozogamicin; IDA = idarubicine; ICE = (idarubicin 12 mg/m^2^ i.v. days 1 and 3, cytarabine 100 mg/m^2^ cont. i.v. days 1–5, etoposide 100 mg i.v. days 1 and 3); HAM = cytarabine 0.5 g/m^2^/12 h i.v., days 1–3; mitoxantrone 10 mg/m^2^ i.v., days 2 and 3; IEiv = idarubicin 12 mg/m^2^ i.v. days 1 and 3, etoposide 100 mg/m^2^ i.v. days 1–5; IEpo = idarubicin 5mg p.o. days 1, 4, 7, 10, 13; etoposide 100mg p.o. days 1 and 13; repeated on day 29 for 12 courses; DAT = Daunorubicin 50 or 35 mg/m^2^ IV days 1, 2, 3 IV, Cytosine Arabinoside 100 or 200 mg/m2 IV 12-hourly on day 1–10, Thioguanine 100 mg/m2 oral 12-hourly days 1–10; MIDAC = Mitoxantrone 8 mg/m2 IV on days 1, 2 and 3 Cytosine Arabinoside 500 mg/m^2^ by 2-h infusion 12-hourly, days 1, 2 and 3.

**Table 3:** Post-remission therapy in elderly AML.

Group	Study	Post remission Therapy	Outcome
MRC 41	AML-11	1 vs 4 cycles	No difference
HOVON 39	AML-43	GO vs observation	No difference
ALFA 42	9803	6 months non- intensive vs 1 intensive cycle	Better DFS and OS at 2y

**Table 4:** Summary of clinical trials examining azacitidine in monotherapy for untreated WHO-defined acute myeloid leukemia

Study	Patients (n)	AML with 20–30% blasts (n)	Median Age (yrs)	ORR (%)	CR rate (%)	2-year OS (%of patients)	Median survival (months)

Silverman 58,61	91	91	66	36	9	NA	19.3

Fenaux 62	Aza 55	Aza 55	70	NA	18	50	24.5
	CCR 58	CCR 58	70	NA	15 (LDAC), 55 (IC)	16	16.0

Seymour 64	Aza 38	Aza 12	78	58	NA	55	NR
	CCR 49	CCR 18	77	39	NA	15	10.8

Thepot 65	138	44	73	21	14	18	10.2

Maurillo 69	82	16	77	48	19	13	9

Abbreviations: ORR = overall response rate; CR = complete remission; OS = overall survival; Aza = azacitidine; CCR = common conventional regimens; LDAC = low dose cytarabine; IC = intensive chemotherapy; NA = not available; NR = not reached

**Table 5:** Summary of clinical trials examining decitabine in monotherapy for untreated WHO-defined acute myeloid leukemia

Study	Patients (n)	Regimen	AML with 20–30% blasts (n)	Median Age (yrs)	ORR (%)	CR rate (%)	Median survival (months)

Cashen 80	55	20 mg/m^2^/day for 5 days every 4 wks	18	74	25	28	7.7

Blum 81	53	20 mg/m^2^/day for 10 days followed by 4–5 day cycles every 4 wks	13	74	64	47	12.7

Kantarjian 82	238	20 mg/m^2^/day for 5 days every 4 wks vs SC or LDAC	65	73	20.3	18	7.7
	237		58	73	11.5	8	5

Abbreviations: ORR = overall response rate; CR = complete remission; SC = supportive care; LDAC = low dose cytarabine; wks = weeks

## References

[1] Bennett JM, Catovsky D, Daniel MT, Flandrin G, Galton DAG, Gralnick HR (1982). Proposals for the classification of the myelodysplastic syndromes. Br J Haematol.

[2] Sanz GF, Sanz MA, Vallespi T, Canizo MC, Torrabadella M, Garcia S (1989). Two regression models and a scoring system for predicting survival and planning treatment in myelodysplastic syndromes: a multivariate analysis of prognostic factors in 370 patients. Blood.

[3] Greenberg P, Cox C, LeBeau MM, Fenaux P, Morel P, Sanz G (1997). International scoring system for evaluating prognosis in myelodysplastic syndromes. Blood.

[4] Germing U, Gattermann N, Strupp C, Aivado M, Aul C (2000). Validation of the WHO proposals for a new classification of primary myelodysplastic syndromes: a retrospective analysis of 1600 patients. Leuk Res.

[5] Seymour JF, Estey EH (1993). The prognostic significance of Auer rods in myelodysplasia. Br J Haematol.

[6] Seymour JF, Estey EH (1995). The contribution of Auer rods to the classification and prognosis of myeldysplastic syndromes. Leuk Lymphoma.

[7] Vallespi T, Sanz G, Torrabadella M, Irriguible D, Sanz MA (1990). Auer rods in myelodysplastic syndromes (MDS): presence and significance. Blood.

[8] Michels SD, Saumur J, Arthur DC, Robison LL, Brunning RD (1989). Refractory anemia with excess of blasts in transformation: hematologic and clinical study of 52 patients. Cancer.

[9] Harris NL, Jaffe ES, Diebold J (1999). J Clin Oncol.

[10] Albitar M, Beran M, O’Brien S, Kantarjian H, Frierich E, Keating M, Estey E (2000). Differences between refractory anemia with excess blasts in transformation and acute myeloid leukaemia. Blood.

[11] Vardiman JW, Harris NL, Brunning RD (2002). The World Health Organization (WHO) classification of the myeloid neoplasms. Blood.

[12] Orazi A, Bennett JM, Germing U, Brunning RD, Bain BJ, Thiele J, Swerdlow SH, Campo E, Jaffe ES (2008). Chronic myelomonocytic leukaemia. World Health Organization Classification of Tumours of Haematopoietic and Lymphoid Tissues.

[13] Haferlach T, Schoch C, Loffler H (2003). Morphologic dysplasia in de novo acute myeloid leukemia (AML) is related to unfavourable cytogenetics but has no independent prognostic relevance under the conditions of intensive induction therapy: results of a multiparameter analysis from the German AML Cooperative Group studies. J Clin Oncol.

[14] Wandt H, Schakel U, Kroschinsky F (2008). MLD according to the WHO classification in AML has no correlation with age and no independent prognostic relevance as analyzed in 1766 patients. Blood.

[15] Weinberg OK, Seetharam M, Ren L (2009). Clinical characterization of acute myeloid leukemia with myelodysplasia-related changes as defined by the 2008 WHO classification system. Blood.

[16] Arber DA, Brunning RD, Orazi A, Bain BJ, Porwit A, Vardiman JW, Swerdlow SH, Campo E, Harris NL, Jaffe ES, Pileri SA, Stein H (2008). Acute myeloid leukaemia with myelodysplasia-related changes. WHO classification of tumours of haematopoietic and lymphoid tissues.

[17] Miesner M, Haferlach C, Bacher U (2010). Multilineage dysplasia (MLD) in acute myeloid leukemia (AML) correlates with MDS-related cytogenetic abnormalities and a prior history of MDS or MDS/MPN but has no independent prognostic relevance: a comparison of 408 cases classified as AML not otherwise specified (AML-NOS) or “AML with myelodysplasia-related changes” (AML-MRC). Blood.

[18] Voso MT, D’Alo F, Greco M, Fabiani E, Criscuolo M, Migliora G (2010). Epigenetic changes in therapy-related MDS/AML. Chem Biol Interact.

[19] Tien HF, Tang JH, Tsay W, Liu MC, Lee FY, Wang CH (2001). Methylation of the p15(INK4B) gene in myelodysplastic syndrome: it can be detected early at diagnosis or during disease progression and is highly associated with leukaemic transformation. Br J Haematol.

[20] Christiansen DH, Andersen MK, Pedersen-Bjergaard J (2003). Methylation of p15INK4B is common, is associated with deletion of genes on chromosome arm 7q and predicts a poor prognosis in therapy-related myelodysplasia and acute myeloid leukemia. Leukemia.

[21] Abdel Wahab O, Figueroa ME (2012). Interpreting new molecular genetics in myelodysplastic syndrome. Hematology Am Soc Hematol Educ Program.

[22] Shih AH, Abdel Wahab O, Patel JP, Levine RL (2012). The role of mutations in epigenetic regulators in myeloid malignancies. Nat Rev Cancer.

[23] Hwang SS, Scott CB, Chang VT (2004). Prediction of survival for advanced cancer patients by recursive partitioning analysis: Role of Karnofsky performance status, quality of life, and symptom distress. Cancer Invest.

[24] Klepin HD, Geiger AM, Tooze JA (2011). The feasibility of inpatient geriatric assessment for older adults receiving induction chemotherapy for acute myelogenous leukemia. J Am Geriatr Soc.

[25] Juliusson G, Antunovic P, Derolf A (2009). Age and acute myeloid leukemia: real world data on decision to treat and outcomes from the Swedish Acute Leukemia Registry. Blood.

[26] Appelbaum F, Gundacker H, Head DR (2006). Age and acute myeloid leukemia. Blood.

[27] Luger SM (2010). Treating the elderly patient with acute myelogenous leukemia. Hematology Am Soc Hematol Educ Program.

[28] Rowe JM (2009). Closer to the truth in AML. Blood.

[29] Estey E, Thall P, Beran M (1997). Effect of diagnosis (refractory anemia with excess blasts, refractory anemia with excess blasts in transformation, or acute myeloid leukemia [AML]) on outcome of AML-type chemotherapy. Blood.

[30] Bernstein SH, Brunetto VL, Davey FR (1996). Acute myeloid leukemia-type chemotherapy for newly diagnosed patients without antecedent cytopenias having myelodysplastic syndrome as defined by French-American-British criteria: A Cancer and Leukemia Group B study. J Clin Oncol.

[31] Lowenberg B, Ossenkoppele GJ, van Putten W (2009). High-dose daunorubicin in older patients with acute myeloid leukemia. N Engl J Med.

[32] Pautas C, Merabet F, Thomas X (2010). Randomized study of intensified anthracycline doses for induction and recombinant interleukin-2 for maintenance in patients with acute myeloid leukemia age 50 to 70 years: results of the ALFA-9801 study. J Clin Oncol.

[33] Burnett AK, Milligan D, Goldstone A (2009). The impact of dose escalation and resistance modulation in older patients with acute myeloid leukaemia and high risk myelodysplastic syndrome: the results of the LRF AML 14 trial. Br J Haematol.

[34] Cripe LD, Uno H, Paietta EM (2010). Zosuquidar, a novel modulator of P-glycoprotein, does not improve the outcome of older patients with newly diagnosed acute myeloid leukemia: a randomized, placebo-controlled, trial of the Eastern Cooperative Oncology Group (ECOG 3999). Blood.

[35] Burnett A, Russell N, Kell J (2010). European development of clofarabine as treatment for older patients with acute myeloid leukemia considered unsuitable for intensive chemotherapy. J Clin Oncol.

[36] Faderl S, Erba HP, Claxton DF (2009). Clofarabine produces durable remissions in older patients with AML with unfavorable prognostic factors and multiple comorbidities. Blood.

[37] Faderl S, Ravandi F, Huang X (2008). A randomized study of clofarabine versus clofarabine plus low-dose cytarabine as front-line therapy for patients aged 60 years and older with acute myeloid leukemia and high-risk myelodysplastic syndrome. Blood.

[38] Mayer RJ, Davis RB, Schiffer CA (1994). Intensive postremission chemotherapy in adults with acute myeloid leukemia. N Engl J Med.

[39] Lowenberg B, Beck J, Graux C (2010). Gemtuzumab ozogamicin as postremission treatment in AML at 60 years of age or more: results of a multicenter phase 3 study. Blood.

[40] Baer MR, George SL, Caligiuri MA (2008). Low-dose interleukin-2 immunotherapy does not improve outcome of patients age 60 years and older with acute myeloid leukemia in first complete remission: Cancer and Leukemia Group B Study 9720. J ClinOncol.

[41] Goldstone AH, Burnett AK, Wheatley K (2001). Attempts to improve treatment outcomes in acute myeloid leukaemia (AML) in older patients: the results of the United Kingdom Medical Research Council AML11 trial. Blood.

[42] Gardin C, Turlure P, Fagot T (2007). Post remission treatment of elderly patients with acute myeloid leukemia in first complete remission after intensive induction chemotherapy: results of the multicenter randomized Acute Leukemia French Association (ALFA) 9803 trial. Blood.

[43] Schlenk RF, Frohling S, Hartmann F (2006). Intensive consolidation versus oral maintenance therapy in patients 61 years or older with acute myeloid leukemia in first remission: results of second randomization of the AML HD98-B treatment trial. Leukemia.

[44] Chevallier P, Blaise D, Milpied N (2009). Reduced intensity conditioning (RIC) allogeneic stem cell transplantation for patients aged >=60 years: a retrospective study of 629 patients from the Societe Francaise De Greffe De Moelle et de therapie cellulaire (SFGM-TC). Blood.

[45] Röllig C, Thiede C, Gramatzki M (2010). A novel prognostic model in elderly patients with acute myeloid leukemia: results of 909 patients entered into the prospective AML96 trial. Blood.

[46] Estey E, de Lima M, Tibes R (2007). Prospective feasibility analysis of reduced-intensity conditioning (RIC) regimens for hematopoietic stem cell transplantation (HSCT) in elderly patients with acute myeloid leukemia (AML) and high-risk myelodysplastic syndrome (MDS). Blood.

[47] Kurosawa S, Yamaguchi T, Uchida N (2011). Comparison of allogeneic hematopoietic cell transplantation and chemotherapy in elderly patients with non-M3 acute myelogenous leukemia in first complete remission. Biol Blood Marrow Transplant.

[48] Estey E (2007). Acute myeloid leukemia and myelodysplastic syndromes in older patients. J Clin Oncol.

[49] Kantarjian H, Ravandi F, O’Brien S (2010). Intensive chemotherapy does not benefit most older patients (age 70 years or older) with acute myeloid leukaemia. Blood.

[50] Issa JP, Baylin SB, Herman JG (1997). DNA methylation changes in hematologic malignancies: biologic and clinical implications. Leukemia.

[51] Desmond JC, Raynaud S, Tung E, Hofmann W-K, Haferlach T, Koeffler HP (2007). Discovery of epigenetically silenced genes in acute myeloid leukemias. Leukemia.

[52] Redner RL, Wang J, Liu JM (1999). Chromatin remodeling and leukemia: new therapeutic paradigms. Blood.

[53] Will B, Zhou L, Vogler TO (2012). Stem and progenitor cells in myelodysplastic syndromes show aberrant stage-specific expansion and harbor genetic and epigenetic alterations. Blood.

[54] Jones PA, Laird PW (1999). Cancer epigenetics comes of age. Nat Genet.

[55] Leone G, Teofili L, Voso MT (2002). DNA methylation and demethylating drugs in myelodysplastic syndromes and secondary leukemias. Hematologica.

[56] Santini V, Kantarjian HM, Issa JP (2001). Changes in DNA methylation in neoplasia: pathophysiology and therapeutic implications. Ann Intern Med.

[57] Silverman LR (2001). Oncologist.

[58] Silverman LR, Holland JF, Demakos EP (1994). Azacitidine (AZA C) in myelodysplastic syndromes (MDS), CALGB studies 8421 and 8921. [abstr46]. Ann Hematol.

[59] Silverman LR, Demakos EP, Peterson BL (2002). Randomized controlled trial of azacitidine inpatients with the myelodysplastic syndrome: a study of the Cancer and Leukemia Group B. J Clin Oncol.

[60] Cheson BD, Bennett JM, Kantarjian H (2000). Report of an international working group to standardize response criteria for myelodysplastic syndromes. Blood.

[61] Silverman LR, McKenzie DR, Peterson BL, Holland JF, Backstrom JT, Beach CL (2006). Further analysis of trials with azacitidine in patients with myelodysplastic syndrome: studies 8421, 8921, and 9221 by the Cancer and Leukemia Group B. J Clin Oncol.

[62] Fenaux P, Mufti GJ, Hellstrom-Lindberg E, Santini V, Finelli C, Giagounidis A (2009). Efficacy of azacitidine compared with that of conventional care regimens in thetreatment of higher-risk myelodysplastic syndromes: a randomised, open-label, phase III study. Lancet Oncol.

[63] Fenaux P, Mufti GJ, Hellström-Lindberg E (2009). Azacitidine prolongs overall survival compared with conventional care regimens in older adult patients with low bone marrow blast count 20–30 %) acute myeloid leukemia. J Clin Oncol.

[64] Celgene Corporation Study of Vidaza versus conventional care regimens for the treatment of acute myeloid leukemia (AML) [ClinicalTrialsgov identifier NCT01074047]. US National Institutes of Health Clinical Trials.gov [online].

[65] Seymour JF, Fenaux P, Silverman LR (2010). Effects of azacitidine compared with conventional care regimens in elderly( ≥75 years) patients with higher-risk myelodysplastic syndromes. Crit Rev Oncol Hematol.

[66] Thépot S, Itzykson R, Seegers V (2009). Azacytidine(AZA) as first line therapy in AML: results of the French ATU program. Blood (ASH Annual MeetingAbstracts).

[67] Itzykson R, Thépot S, Recher C (2009). Azacytidine in refractory or relapsed AML after intensive chemotherapy (IC): results of the French ATU program. Blood (ASH Annual Meeting Abstracts).

[68] Itzykson R, Thépot S, Quesnel B (2011). Prognostic factors for response and overall survival in 282 patients with higher-risk myelodysplastic syndromes treated with azacitidine. Blood.

[69] Maurillo L, Venditti A, Spagnoli A (2012). Azacitidine for the treatment of patients with acute myeloid leukemia: report of 82 patients enrolled in an Italian Compassionate Program. Cancer.

[70] Al Ali HK, Jaekel N, Junghanss C (2012). Azacitidine in patients with acute myeloid leukemia medically unfit for or resistant to chemotherapy: a multicenter phase I/II study. Leuk Lymphoma.

[71] Van der Helm LH, Alhan C, Wijermans PW (2011). Platelet doubling after the first azacitidine cycle is a promising predictor for response in myelodysplastic syndromes (MDS), chronic myelomonocytic leukaemia (CMML) and acute myeloid leukemia (AML) patients in the Dutch azacitidine compassionate named patient programme. Br J Haematol.

[72] Soriano AO, Yang H, Faderl S (2007). Safety and clinical activity of the combination of 5-azacytidine, valproic acid, and all-trans retinoic acid in acute myeloid leukemia and myelodysplastic syndrome. Blood.

[73] Raffoux E, Cras A, Recher C (2010). Phase 2 clinical trial of 5-azacitidine, valproic acid, and all-trans retinoic acid in patients with high-risk acute myeloid leukemia or myelodysplastic syndrome. Oncotarget.

[74] Raza A, Mehdi M, Mumtaz M, Ali F, Lascher S, Galili N (2008). Combination of 5-azacytidine and thalidomide for the treatment of myelodysplastic syndromes and acute myeloid leukemia. Cancer.

[75] Silverman LR, Verma A, Odchimar-Reissig R (2008). A phase I/II study of vorinostat, an oral histone deacetylase inhibitor, in combination with azacitidine in patients with the myelodysplastic syndrome (MDS) and acute myeloid leukaemia (AML). Initial results of the phase I trial: a New York Cancer Consortium. J Clin Oncol.

[76] Pollyea DA, Zehnder J, Coutre S (2012). Sequential azacitidine plus lenalidomide combination for elderly patients with untreated acute myeloid leukaemia. Haematologica.

[77] Grovdal M, Karimi M, Khan R (2010). Manteinance treatment with azacitidine for patients with high risk myelodysplastic syndromes (MDS) or acute myeloid leukemia following MDS in complete remission after induction therapy. Br J Haematol.

[78] Gardin C, Prébet T, Bouabdallah K (2009). A phase II study of post-remission therapy with azacitidine (AZA) in patients with AML post-MDS and high risk MDS: a GFM group study. Blood (ASH Annual Meeting Abstracts).

[79] Christman JK (2002). 5-Azacytidine and 5-aza-2-deoxycytidine as inhibitors of DNA methylation: mechanistic studies and their implications for cancer therapy. Oncogene.

[80] Cashen AF, Schiller GJ, O’Donnell MR (2010). Multicenter, phase II study of decitabine for the first-line treatment of older patients with acute myeloid leukemia. J Clin Oncol.

[81] Blum W, Garzon R, Klisovic RB (2010). Clinical response and miR-29b predictive significance in older patients treated with a 10 day schedule of decitabine. Proc Natl Acad Sci.

[82] Kantarjian HM, Thomas XG, Dmoszynska A (2012). Multicenter, randomized, open-label, phase III trial of decitabine versus patient choice, with physician advice, of either supportive care or low-dose cytarabine for the treatment of older patients with newly diagnosed acute myeloid leukaemia. J Clin Oncol.

[83] Blum W, Klisovic RB, Hackanson B (2007). Phase 1 study of decitabine alone or in combination with valproic acid in acute myeloid leukemia. JClin Oncol.

[84] Kirshbaum M, Gojo I, Goldber SL (2009). Vorinostat in combination with decitabine for the treatment of relapsed or newly diagnosed acute myelogenous leukemia (AML) or myelodysplastic syndrome (MDS): a Phase 1 dose escalation study. Blood.

